# Evaluation of the Tier 1 Program of Project P.A.T.H.S.: Secondary Data Analyses of Conclusions Drawn by the Program Implementers

**DOI:** 10.1100/tsw.2008.6

**Published:** 2008-01-14

**Authors:** Daniel T. L. Shek

**Affiliations:** ^1^ Centre for Quality of Life, Hong Kong Institute of Asia-Pacific Studies, The Chinese University of Hong Kong, Hong Kong; ^2^ Social Welfare Practice and Research Centre, The Chinese University of Hong Kong, Hong Kong; ^3^ Kiang Wu Nursing College of Macau, Macau

**Keywords:** positive youth development, subjective outcome evaluation, secondary data analysis, Chinese adolescents

## Abstract

The Tier 1 Program of the Project P.A.T.H.S. (Positive Adolescent Training through Holistic Social Programmes) is a curricula-based positive youth development program. In the experimental implementation phase, 52 schools participated in the program. Based on subjective outcome evaluation data collected from the program participants (Form A) and program implementers (Form B) in each school, the program implementers were invited to write down five conclusions based on an integration of the evaluation findings (N = 52). The conclusions stated in the 52 evaluation reports were further analyzed via secondary data analyses in this paper. Results showed that most of the conclusions concerning perceptions of the Tier 1 Program, instructors, and effectiveness of the programs were positive in nature. There were also conclusions reflecting the respondents’ appreciation of the program. Finally, responses on the difficulties encountered and suggestions for improvements were observed. In conjunction with the previous evaluation findings, the present study suggests that the Tier 1 Program was well received by the stakeholders and the program was beneficial to the development of the program participants.

### INTRODUCTION

In the literature of evaluation, there are different views regarding the role played by the program implementers in the evaluation process[1]. In the traditional experimental approach, since program implementers are not the "recipients" of the program, their views are either immaterial or such information is at best regarded as "noise" variables that may confound the outcomes. In essence, objective outcome findings rather than subjective outcome findings should be the focus. In the subjective outcome evaluation approach (i.e., client satisfaction approach), although views of the participants are the ultimate concern, it is not a common practice to examine the views of the program implementers. In the qualitative evaluation paradigm, the views of different parties are commonly assessed to reveal the complexity of the phenomena under investigation. In fact, it would be incomplete if the program implementers' views are left out. In the utilization-focused evaluation paradigm, as different stakeholders are involved in the evaluation process, program implementers' views are legitimately covered. In addition, flexible evaluation designs in response to different situations should be developed. Finally, based on the standards of the Joint Committee on Standards for Educational Evaluation[2], identification of the stakeholders (Utility Standard 1) involving complete and fair assessment (Proprietary Standard 5) would be important. According to these standards, program implementers' views and assessment should be taken into account.

Nevertheless, there are several arguments against the use of evaluation data collected from the program implementers. The first argument is that program implementers may not have the required expertise in conducting evaluation. Second, there may be role strain and role confusion involved if program implementers have to perform the roles of both program implementer and evaluator. Third, there are several sources of bias that are involved in the evaluation conducted by program implementers. In the first place, since program implementers have to be accountable for their delivered service, they may boost the effectiveness for the sake of job security (i.e., rice-bowl argument). In addition, since program implementers have invested time and effort in the program implementation process, it is difficult for them to evaluate a program in a negative manner (i.e., cognitive dissonance argument). On the other hand, as the program implementers may not be totally willing to implement a program, they may consciously or unconsciously minimize the program effectiveness and evaluate the program in an unfavorable manner (i.e., revenge argument).

On the other hand, there are several counter-arguments with reference to the above criticisms of involving program implementers in the evaluation process. First, some professionals, such as teachers and social workers, are trained to conduct evaluation research. Second, since evaluation is part of practice in many professions, professionals are actually expected to implement the program as well as to evaluate the program. In the case of teachers and social workers, role conflict is basically not a problem. In fact, they are expected to carry out both program implementation and evaluation tasks in their practice. In addition, the emphasis on reflective practice in these professions actually encourages professionals to evaluate the delivered programs in an honest and sincere manner. Third, based on different evaluation perspectives (e.g., qualitative evaluation, illuminative evaluation, utilization-focused evaluation), it is legitimate and indispensable to collect the views of the program implementers (conservative view) or to engage the program implementers as evaluators (liberal view).

Evaluation studies utilizing the views and conclusions reached by colleagues in the field can be seen in the literature. For example, as reported by Miles and Huberman[3], Stearns et al.[4] studied 22 schools in which a new special education law had been implemented. For each site, the field workers prepared case study reports. As there were many reports and the data were voluminous, the researchers attempted to describe commonalities and differences across sites, and prepare conclusions for 22 sites based on the reports of the field workers through seven steps:

- Step 1: Each field worker was invited to develop a list of statements he or she "would like to see in the final report", which could be unstructured in nature.
- Step 2: The 1,500 unstructured statements initially derived were reduced to 1,000 by discarding the duplicated statements.
- Step 3: From the 1,000 statements, the general and abstract statements were retained (N = 250).
- Step 4: The 250 statements were sorted into 30 general categories with reference to "assumptions", "findings", and "conclusions". The analysts further examined the inter-relationships among these 30 categories of statements.
- Step 5: Based on the findings, 250 propositions organized around 21 headings were then developed and presented to the field workers for review.
- Step 6: Field workers examined the validity and relevance of each proposition.
- Step 7: The researchers wrote a "findings report" for each of the 21 topics with reference to the different sites.

To promote holistic development among adolescents in Hong Kong, the Hong Kong Jockey Club Charities Trust approved HK$400 million to launch a project entitled “P.A.T.H.S. to Adulthood: A Jockey Club Youth Enhancement Scheme”. “P.A.T.H.S.” denotes Positive Adolescent Training through Holistic Social Programmes[5,6]. There are two tiers of programs (Tier 1 and Tier 2) in this project. The Tier 1 Program is a universal positive youth development program where students in Secondary 1 to 3 were invited to participate in the program, normally with 20 h of training in the school year at each grade. After completion of the Tier 1 Program, program participants and program implementers were required to complete subjective outcome evaluation forms (Form A and Form B, respectively). Based on the subjective outcome evaluation data collected in each school, the responsible worker was required to complete an evaluation report, where they were asked to write down five conclusions regarding the program and its effectiveness. In this study, secondary data analyses were carried out to examine the conclusions drawn by the workers based on the subjective outcome evaluation findings.

### METHODS

#### Dataset for Secondary Data Analyses

There were 52 schools that joined the Experimental Implementation Phase. After the Tier 1 Program was completed, the participants were invited to respond to a subjective outcome evaluation questionnaire. A total of 8,057 students (mean = 154.94 students per school, range: 37–212) responded to the Subjective Outcome Evaluation Form (Form A)[7,8,9,10]. Similarly, the workers were invited to respond to a subjective outcome evaluation questionnaire after the completion of the program. A total of 344 workers responded to the Subjective Outcome Evaluation Form (Form B)[7,8,9,10].

Form A was designed by Shek and Siu[see 7,8,9,10]. Broadly speaking, there are several parts in this evaluation form, including (a) participants’ perceptions of the program, such as program objectives, design, classroom atmosphere, interaction among the students, and the respondent’s participation during class (10 items); (b) participants’ perceptions of the workers, such as the preparation of the instructor, professional attitude, involvement, and interaction with the students (10 items); (c) participants’ perception of the effectiveness of the program, such as promotion of different psychosocial competencies, resilience, and overall personal development (16 items); (d) the extent to which the participant would recommend the program to other people with similar needs (1 item); (e) the extent to which the participant would join similar programs in future (1 item); (f) overall satisfaction with the program (1 item); (g) things that the participant learned from the program (opened-ended question); (h) things that the participant appreciated most (open-ended question); (i) opinion about the instructor(s) (open-ended question); and (j) areas that require improvement (open-ended question).

Form B was also designed by Shek and Siu[see 7,8,9,10]. Broadly speaking, there are several parts in this evaluation form, including (a) program implementers’ perceptions of the program, such as program objectives, design, classroom atmosphere, interaction among the students, and the respondent’s participation during class (10 items); (b) program implementers’ perceptions of their own practice, including their understanding of the course, teaching skills, professional attitude, involvement, and interaction with the students (10 items); (c) workers’ perceptions of the effectiveness of the program, such as promotion of different psychosocial competencies, resilience, and overall personal development (16 items); (d) the extent to which the worker would recommend the program to other students with similar needs (1 item); (e) the extent to which the worker would teach similar programs in future (1 item); (f) overall satisfaction with the program (1 item); (g) things that the worker obtained from the program (opened-ended question); (h) things that the worker appreciated most (open-ended question); (i) difficulties encountered (open-ended question); and (j) areas that require improvement (open-ended question).

Based on the evaluation data collected in each school, the responsible worker in each school was required to complete an Evaluation Report where the quantitative and qualitative findings based on Form A and Form B were summarized and described. In the last section of the report, the worker preparing the report in each school was requested to write down five conclusions regarding the program and its effectiveness, which can give a holistic picture regarding the perceived effectiveness of the Tier 1 Program.

### Data Analyses

The data were analyzed using general qualitative analyses techniques[3] by several research assistants. There were three steps in the data analysis process. First, raw codes were developed for words, phrases, and/or sentences that formed meaningful units in each conclusion at the raw responses level. Second, the codes were further combined to reflect higher-order attributes at the category of codes level. For example, the response of “the program is helpful to students” at the raw response level could be subsumed under the category of “general program benefit”, which could be further subsumed under the broad theme of “benefits of the program” (see Table 1).

In the present qualitative analyses, since the author designed the P.A.T.H.S. program, he was conscious of his own biases and expectation of the program to be effective. As such, the author was not directly involved in the data analyses. In addition, in order to minimize the possible biases involved, both intra- and inter-rater reliability on the coding was calculated. For intrarater reliability, the research assistant primarily responsible for coding coded 20 randomly selected responses without looking at the original codes given. For inter-rater reliability, another research assistant coded 20 randomly selected responses without knowing the original codes given at the end of the scoring process.

Following the principles of qualitative analyses proposed by Shek et al.[11], the following attributes of the study regarding data collection and analyses are highlighted. First, a general qualitative orientation was adopted. Second, the sources of data (e.g., number of participants) for analyses are described. Third, the issues of biases and ideological preoccupation are addressed. Fourth, inter- and intrarater reliability information is presented. Fifth, the categorized data were kept by a systematic filing system in order to ensure that the findings are auditable. Finally, possible explanations, including alternative explanations, are considered.

## RESULTS

Based on the 257 conclusions in the 52 reports (two schools gave three conclusions and one school gave six conclusions), 470 meaningful units were extracted. These raw responses were further categorized into several categories, including views of the stakeholders on the program (Table 1), views of stakeholders on the program implementers (Table 2), perceived general and specific benefits of the program (Table 3), difficulties encountered (Table 4), areas appreciated by the stakeholders (Table 5), suggestions for improvement (Table 6), and other responses (Table 7).

Regarding the conclusions related to the stakeholders’ perceptions of the program, results in Table 1 showed that most of the responses were positive in nature in the areas of satisfaction, program content, and program arrangement. Among the 109 responses, 93 responses were classified as positive (85.32%). The intra-rater agreement percentage was 100% and inter-rater agreement percentage was 83%. For the perceptions of the program implementers, findings in Table 2 also showed that most of the responses were positive in nature. Among the 65 responses, 64 were positive (98.46%).

The responses related to perceived general and specific benefits of the program to the students are shown in Table 3. There were a total of 170 meaningful units that could be categorized in several categories, including perceived general effectiveness and perceived specific effectiveness (connection, competence, confidence, character, caring/contribution, others). Overall, the positive effects of the program were evident. Among 170 responses, 158 were positive (92.94%). The intra-rater agreement percentage was 100% and inter-rater agreement percentage was 93%.

**TABLE 1**
**Responses Related to the Stakeholders' Views on the Program**

| Category | Responses | Nature of the Response | | | | Total |
|---|---|---|---|---|---|---|
| | | Positive | Neutral | Negative | Undecided | |
| Satisfaction level | Liked the program very much | 2 | | | | 2 |
| | Liked the program | 6 | | | | 6 |
| | Quite liked the program | 1 | | | | 1 |
| | Satisfied with the program | 35 | | | | 35 |
| | Satisfied with the program with comment | 3 | | | | 3 |
| | Acceptable | 1 | | | | 1 |
| | Positive comment | 7 | | | | 7 |
| | Enjoyed the program | 1 | | | | 1 |
| | Negative comment | | | 2 | | 2 |
| | **Subtotal** | **56** | **0** | **2** | **0** | **58** |
| Program content | Interactive | 10 | | | | 10 |
| | Good atmosphere | 2 | | | | 2 |
| | Liked the games | 1 | | | | 1 |
| | Diversified | 1 | | | | 1 |
| | Systematic | 2 | | | | 2 |
| | Experiential learning | | | | 1 | 1 |
| | Appreciated the activities | 3 | | | | 3 |
| | Interesting | 3 | | | | 3 |
| | Comprehensive content | 2 | | | | 2 |
| | Detailed information | 1 | | | | 1 |
| | Teaching material not boring | 2 | | | | 2 |
| | Infeasible content | | | 1 | | 1 |
| | Match student needs | 2 | | | | 2 |
| | Too many worksheets | | | 2 | | 2 |
| | Satisfied with objective and theory of the program | 4 | | | | 4 |
| | Lack of interest | | | 1 | | 1 |
| | Negative view towards content | | | 3 | | 3 |
| | Need to be improved | | | 4 | | 4 |
| | **Subtotal** | **33** | **0** | **11** | **1** | **45** |
| Program arrangement | Satisfied | 1 | | | | 1 |
| | Time constraint | | | 2 | | 2 |
| | Systematic | 1 | | | | 1 |
| | Whole school cooperation | 2 | | | | 2 |
| | **Subtotal** | **4** | **0** | **2** | **0** | **6** |
| **Total responses** | | **93** | **0** | **15** | **1** | **109** |

**TABLE 2**
**Responses on the Views towards Instructors**

| Category | Responses | Nature of Response | | | | Total |
|---|---|---|---|---|---|---|
| | | Positive | Neutral | Negative | Undecided | |
| Satisfaction level | Satisfied very much | 3 | | | | 3 |
| | Satisfied | 42 | | | | 42 |
| | Very positive comment | 3 | | | | 3 |
| | Positive comment | 2 | | | | 2 |
| | **Subtotal** | **50** | **0** | **0** | **0** | **50** |
| Views about the instructors | Devoted | 4 | | | | 4 |
| | Professional attitude | 2 | | | | 2 |
| | Earnest attitude | 1 | | | | 1 |
| | Caring attitude | 2 | | | | 2 |
| | Care about student | 1 | | | | 1 |
| | Helping student | 2 | | | | 2 |
| | Mastery of teaching skill | 1 | | | | 1 |
| | Instructor's attitude and performance influenced students' learning | | | | 1 | 1 |
| | Good program arrangement | 1 | | | | 1 |
| | **Subtotal** | **14** | **0** | **0** | **1** | **15** |
| **Total responses** | | **64** | **0** | **0** | **1** | **65** |

The difficulties encountered are presented in Table 4. It is noteworthy that not many responses were abstracted from the conclusions (N = 21). The responses reflecting appreciation of the program can be seen in Table 5. The suggestions for improvement can be seen in Table 6 (N = 75). It is noteworthy that some suggestions for improvement are contradictory (e.g., increase the number of units vs. decrease the number of units). Finally, responses not captured in the preceding tables can be seen in Table 7 (N = 21).

As it is difficult to determine whether the responses related to suggestions for improvement (Table 6) were really negative responses or not, it may be possible to discard them when calculating the percentage of positive responses. Based on this approach, the overall proportion of positive responses was 85.06%. However, even if the responses in Table 6 were included in the analyses, the percentage of positive responses was still 71.49%. To further examine the reliability of the analyses, 20 responses were randomly selected from all responses in Table 1 to Table 7 for checking. Analyses showed that intra-rater agreement percentage was 100% and inter-rater agreement percentage was 85%. These findings suggest that the reliability of coding was on the high side in this study.

### DISCUSSION

Utilizing secondary data analyses, this study attempted to analyze the conclusions drawn by the workers who implemented the P.A.T.H.S. Tier 1 Program. Results showed that most of the responses related to perceptions of the Tier 1 Program, instructors, and effectiveness of the programs were positive in nature. There were also conclusions reflecting the respondents' appreciation of the program. These findings are generally consistent with several lines of evidence supporting the effectiveness of P.A.T.H.S. First, evaluation findings based on objective outcome evaluation showed that there were positive changes in the program participants after joining the program[12]. Second, quantitative as well as qualitative subjective outcome evaluation findings based on program participants and implementers showed that the program

**TABLE 3**
**Responses on Perceived Effectiveness**

| Category | Responses | Nature of Response | | | | Total |
|---|---|---|---|---|---|---|
| | | Positive | Neutral | Negative | Undecided | |
| Perceived general effectiveness | Can help students' development | 33 | | | | 33 |
| | Has some effect on students' development | 5 | | | | 5 |
| | May help students' development | 1 | | | | 1 |
| | Has limited effectiveness to students' development | | | 1 | | 1 |
| | Not sure whether it can help students' development | | | 2 | | 2 |
| | No help to student's development | | | 1 | | 1 |
| | Has negative view toward project effectiveness | | | 1 | | 1 |
| | Can help instructors' development | 2 | | | | 2 |
| | **Subtotal** | **41** | **0** | **5** | **0** | **46** |
| Perceived specific effectiveness | | | | | | |
| Connection | Improved interpersonal relationship | 2 | | | | 2 |
| | Enhanced teacher-student relationship | 6 | | | | 6 |
| | Enhanced peer relationship | 3 | | | | 3 |
| | **Subtotal** | **11** | **0** | **0** | **0** | **11** |
| Competence | Promoted emotional control | 6 | | | | 6 |
| | Unable to improve emotional control | | | 1 | | 1 |
| | Promoted analytical ability | 4 | | | | 4 |
| | Promoted social ability | 14 | | | | 14 |
| | Promoted presentation ability | 2 | | | | 2 |
| | Promoted cognitive ability | 2 | | | | 2 |
| | Promoted communication skill | 1 | | | | 1 |
| | Promoted ability of cooperation with other | 1 | | | | 1 |
| | Promoted ability of differentiation right and wrong | 5 | | | | 5 |
| | Helped to respect others | 1 | | | | 1 |
| | Strengthened positive value | 1 | | | | 1 |
| | **Subtotal** | **37** | **0** | **1** | **0** | **38** |
| Confidence | Enhanced self-confidence | 8 | | | | 8 |
| | Enhanced self-determination ability | 3 | | | | 3 |
| | **Subtotal** | **11** | **0** | **0** | **0** | **11** |
| Character | Promoted self-understanding | 8 | | | | 8 |
| | Helped students to treasure life | 2 | | | | 2 |
| | Promoted reflection of life | 1 | | | | 1 |
| | Enhanced self image | 1 | | | | 1 |
| | Promoted patient | 1 | | | | 1 |
| | Promoted reflection ability | 2 | | | | 2 |
| | **Subtotal** | **15** | **0** | **0** | **0** | **15** |

**TABLE 3 (continued)**

| | | | | | | |
|---|---|---|---|---|---|---|
| Caring/Contribution | Less effective in enhancing care of community | | | 1 | | 1 |
| | **Subtotal** | **0** | **0** | **1** | **0** | **1** |
| Others | Student could gain extracurricula knowledge | 1 | | | | 1 |
| | Enhanced students' participation in classroom | 1 | | | | 1 |
| | **Subtotal** | **2** | **0** | **0** | **0** | **2** |
| **Total responses** | | **117** | **0** | **7** | **0** | **124** |

**TABLE 4**
**Responses on Difficulties Encountered during Program Implementation**

| Category | Responses | Nature of Response | | | | Total |
|---|---|---|---|---|---|---|
| | | Positive | Neutral | Negative | Undecided | |
| Difficulties related to program content | Too much content led to overrun | | | 5 | | 5 |
| | Too many worksheets | | | 1 | | 1 |
| | Lack of flexibility | | | 1 | | 1 |
| | Overlapping of content material | | | 2 | | 2 |
| | Not suitable for non-Chinese students | | | | 1 | 1 |
| | The content is too much to arouse students' bad feeling | | | 1 | | 1 |
| | Too much information, which leads to difficulty in making choice | | | 1 | | 1 |
| | Cannot suit the needs of different schools | | | 1 | | 1 |
| | Insufficient communication between instructor and student | | | 1 | | 1 |
| | Need much time for discussion that affects the learning effect | | | 1 | | 1 |
| | Spent much time for preparing the lessons | | | 1 | | 1 |
| | **Subtotal** | **0** | **0** | **15** | **1** | **16** |
| Difficulties related to program arrangement | Time constraint | | | 2 | | 2 |
| | **Subtotal** | **0** | **0** | **2** | **0** | **2** |
| Other difficulties | No effective marking standard | | | 1 | | 1 |
| | Too much written work that caused overload for the students | | | 1 | | 1 |
| | Difficult in maintaining both teaching and students' discipline | | | 1 | | 1 |
| | **Subtotal** | **0** | **0** | **3** | **0** | **3** |
| **Total responses** | | **0** | **0** | **20** | **1** | **21** |

**TABLE 5**
**Responses Reflecting the Appreciation of the Stakeholders**

| Category | Item | Nature of Response | | | | Total |
|---|---|---|---|---|---|---|
| | | Positive | Neutral | Negative | Undecided | |
| Appreciation of the program | Suggest friend to join the program | 2 | | | | 2 |
| | Would join the program again | 2 | | | | 2 |
| | Teacher training was very good | 1 | | | | 1 |
| | Can help teacher | 2 | | | | 2 |
| | Increase the talking-points with the adolescent | 1 | | | | 1 |
| | Has prevention effect for adolescent | 1 | | | | 1 |
| **Total N** | | **9** | **0** | **0** | **0** | **9** |

participants and implementers had positive perceptions of the program and they generally felt that the program was beneficial to the program participants[7,8,9,10]. Third, convergence between subjective outcome evaluation findings and objective outcome evaluation findings was observed, with those perceiving higher benefits of the program showing greater positive changes[13]. Fourth, qualitative findings based on focus group interviews showed that the program participants had positive views about the program and its associated benefits[14]. Fifth, interim evaluation based on random samples of schools and social work agencies showed that the participants had positive perceptions of the program and its benefits, although they also experienced difficulties and problems[15]. Sixth, process evaluation based on systematic observations showed that the quality of implementation and program adherence were high[16]. Finally, based on students' weekly dairies, students perceived that the program helped them in many areas and the participants generally enjoyed the program[17].

On the other hand, difficulties and suggestions for improvement were observed. The suggestions were generally related to the program content, format, arrangement, and implementation. These findings are consistent with the previous findings based on the interim evaluation and subjective outcome evaluation. As the Tier 1 Program is a curricula-based positive youth development program, it demands that the program implementers adopt a flexible and reflective approach in program implementation. In addition, the teachers are also expected to have much interaction with the students via structured activities, such as role plays and self-disclosure. Because Chinese teachers typically adopt authoritarian roles rather than egalitarian roles in relating to students, teachers might find it difficult to play and share with the students. Furthermore, as the project was launched in the first year on an experimental basis, difficulties and problems were expected. Constructively speaking, the problems encountered and suggestions for improvement can serve as useful pointers to fine tune the program. Nevertheless, it is noteworthy that there were contradictory responses on the program content (e.g., increase the number of lessons vs. decrease the number of lessons; simplify units vs. deepen units), which suggest that there are different views on how the program could be improved.

As far as the evaluation methodology is concerned, the present study made use of the evaluation reports prepared by the program implementers via secondary data analyses. There are several unique features of this approach. First, since evaluation is part of practice, program implementers undertook the evaluation (i.e., program implementers as internal evaluators). Second, the internal evaluator(s) utilized and integrated the subjective outcome evaluation data collected from the program participants and implementers. Third, based on the premise that views of the program implementers should be respected and taken into account, the reports prepared by the workers were used as the primary data.

**TABLE 6**
**Responses on the Recommendations**

| Category | Item | N | N in Category |
|---|---|---|---|
| Content | Need more diversified content | 1 | 26 |
| | Interesting elements should be added | 6 | |
| | Content should be more attractive | 1 | |
| | Improve content of teaching materials | 3 | |
| | Decrease number of units | 1 | |
| | Decrease number of constructs | 2 | |
| | Increase number of lessons | 1 | |
| | Simplify the content | 3 | |
| | Increase more topics related to interpersonal relationships | 1 | |
| | Deepen content | 2 | |
| | Meet students' interests | 3 | |
| | Content should be more lively | 1 | |
| | Match up content and students' ability | 1 | |
| Format | Add more activities | 1 | 32 |
| | Add more games | 2 | |
| | Add more visits | 1 | |
| | Need more diversified format | 5 | |
| | Increase flexibility | 2 | |
| | Arrangement needs to be improved | 1 | |
| | Add more interactive format | 2 | |
| | Prolong discussion and sharing time | 1 | |
| | Add more videos | 1 | |
| | Improve growth puzzles | 2 | |
| | Improve the content of Power Point and worksheet | 1 | |
| | Share real cases | 1 | |
| | Better match of content and time | 12 | |
| Time | Regulate activity time | 2 | 2 |
| Program Implementation | Enhance class discipline | 1 | 15 |
| | Consolidate teaching experience systematically | 2 | |
| | Expand room for amendment | 2 | |
| | Prolong duration of lessons | 4 | |
| | Print student handbook | 1 | |
| | Conduct school-based program | 2 | |
| | Distribute evenly in the lessons within the school year | 1 | |
| | Decrease intensity and number of lessons | 1 | |
| | Strengthen communication of instructor in sharing | 1 | |
| **Total responses** | | | **75** |

**TABLE 7**
**Other Responses**

| Category | Item | Nature of Response | | | | Total |
|---|---|---|---|---|---|---|
| | | Positive | Neutral | Negative | Undecided | |
| Views on the learning/teaching process | Mutual influence of performance of students and instructors | | | | 1 | 1 |
| | Performance of students was influenced by instructors | | | | 2 | 2 |
| | Teacher-student relationship strongly affects effectiveness | | | | 2 | 2 |
| | Good atmosphere can help to build teacher-student relationship and enhance motivation of students | 1 | | | | 1 |
| | Instructors' serious attitude affects students' interest on the topic directly | 1 | | | | 1 |
| | Instructors and students have different opinions | | | | 1 | 1 |
| | Very good lesson atmosphere | 2 | | | | 2 |
| | Experiential learning facilitates traditional concept of classroom discipline | | | | 1 | 1 |
| | **Subtotal** | **4** | **0** | **0** | **7** | **11** |
| Performance of students/instructors | Students and teachers participated actively | 2 | | | | 2 |
| | Students participated actively | 3 | | | | 3 |
| | Satisfied with their own performance | 1 | | | | 1 |
| | Much communication among students | 1 | | | | 1 |
| | Instructor placed importance on communication and relationship with students | 1 | | | | 1 |
| | **Subtotal** | **8** | **0** | **0** | **0** | **8** |
| Others | Students not serious in answering questionnaires | | | 1 | | 1 |
| | Students unwilling to answer questionnaires | | | 1 | | 1 |
| | **Subtotal** | **0** | **0** | **2** | **0** | **2** |
| **Total responses** | | **12** | **0** | **2** | **7** | **21** |

Actually, the approach to analyzing the evaluation reports prepared by the program implementers is consistent with several perspectives within the evaluation literature. These perspectives include utilization-focused evaluation (involvement of the stakeholders in evaluation and the evaluator should be a close and interested gatherer of data)[18,19], movement to treat teachers as researchers/evaluators (documentation of teachers' own practice and teachers serve as internal evaluators)[20], authentic assessment (encouragement of innovation and curriculum practice)[20], and empowerment evaluation. In the evaluation literature, the role of "internal evaluator" has been given increasing attention. Kennedy[21] developed an institutional approach to the evaluation of educational technology in which internal evaluators were involved; Cousins et al.[22] provided justifications for collaborative evaluation and showed that evaluators supported a utilization-focused evaluation approach; Shaw and Faulkner[23] discussed practitioner evaluation at work and remarked that "little is known about the character, homogeneity or diversity, outcomes, motives, and practice of this activity" (p. 44).

Although the present findings can be interpreted as evidence supporting the merits and benefits of P.A.T.H.S., several alternative explanations are present. The first alternative explanation is that the findings are due to insufficient evaluation expertise of the program implementers. However, this alternative explanation can be partially dismissed because professional social workers and teachers receive evaluation training in this project, and evaluation is also part of the training for social workers and teachers in Hong Kong. The second alternative explanation is that the findings are due to biases (e.g., cognitive dissonance, rice bowl, and revenge arguments). However, as the conclusions drawn are based on the subjective outcome evaluation findings, an examination of the findings showed that the conclusions are consistent with the subjective outcome evaluation findings. In short, based on the principle of triangulation, an integration of the existing findings showed that there is a consistent picture derived.

There are several limitations of the study. First, because of nature of secondary data analyses, it is not possible to have more interactive collaboration with the program implementers. Second, because the conclusions written by the workers were brief, the related findings cannot give us an in-depth understanding of the implementation processes involved. Third, validity of the methodology of this study rests on the assumption that program implementers can make reasonable and fair judgments about the program based on the subjective outcome evaluation findings. While this assumption might be met because teachers and social workers are trained to conduct practice evaluation in Hong Kong, inexperienced workers may have difficulties in integrating the subjective outcome evaluation findings and translating them into meaningful conclusions. Despite these limitations and in conjunction with the previous research findings described above, the existing research findings suggest that there are positive perceptions and benefits associated with the P.A.T.H.S. Tier 1 Program in Hong Kong.

### ACKNOWLEDGMENTS

The preparation for this paper and the Project P.A.T.H.S. were financially supported by the Hong Kong Jockey Club Charities Trust.
